# Are clinical measures influenced by various ethnic origins in Iranian patients with ankylosing spondylitis?A pilot study

**Published:** 2014

**Authors:** Sasan Fallahi, Ahmad Reza Jamshidi, Mahdi Mahmoudi, Mostafa Qorbani

**Affiliations:** 1 Internal Medicine Division, Baharloo Hospital, Tehran University of Medical Sciences, International Campus (TUMS-IC),Tehran, Iran.; 2 Rheumatology Research Center, Shariati Hospital, Tehran University of Medical Sciences, Tehran, Iran.; 3 Department of Public Health, Alborz University of Medical Sciences, Karaj, Iran.; 4 Non-Communicable Diseases Research Center, Endocrinology and Metabolism Population Sciences Institute, Tehran University of Medical Sciences, Tehran, Iran.

**Keywords:** Ankylosing spondylitis, Clinical features, Ethnicity, Iranian

## Abstract

***Background: ***Ankylosing spondylitis (AS) may manifest with heterogeneous patterns according to ethnic origins. The purpose of this study was to describe the influence of various Iranian ethnic origins on clinical measures in patients with AS.

***Methods:*** 0ne hundred sixty-three AS patients diagnosed by modified New York 1984 criteria were enrolled consecutively. The patients were classified into Fars, Turk, Kord, Lor and other ethnic origins. Several clinical measures were described and compared between the ethnic origins.

***Results:*** The highest and the lowest finger to floor distance was observed for Fars ethnicity (20.4±14.8) and other ethnicities (5.9±8.1), respectively (P=0.04). The frequency of severe decrease in cervical slope was significantly different between various ethnicities (P=0.025). The most and the least frequency of severe decrease in cervical slope was observed in Fars patients (61.3%) and other ethnicities (20%), respectively. The frequency of severe thoracic kyphosis was significantly dissimilar between various ethnicities (P=0.006). The most and the least frequency of severe increase in thoracic kyphosis was observed in Fars (68.8%) and Lor patients (25%), respectively. A significant relationship was seen only between other ethnicities and finger to floor distance, lateral lumbar flexion, chest expansion and BASDAI (P<0.05).

***Conclusion: ***Clinical expression variations in AS disease might be influenced by various Iranian ethnic origins. A larger sample size with other Iranian ethnicities (Baluch, Arab, etc) is required to clear the definite relationship between Iranian ethnicities and clinical expression.

Ankylosing spondylitis (AS) as a member of the group of spondyloarthropathies is a chronic rheumatic disease with well-known articular and extra-articular manifestations, morbidities and complications (1-3). Spinal mobility restriction is one of the major complications in this disease which may lead to poor functional status, disability, withdrawal from work and increasing healthcare and non-health care costs (4). This complication can be assessed by Metrology indices including Bath AS Metrology Index (BASMI), finger to floor distance, chest expansion, Schober test, etc (5). Some factors such as HLA-B*27, smoking and educational level which influence clinical features have been widely studied in AS (6-10).

Blacks, whites and mixed-race people have been compared in previous studies and some differences have been found between them regarding clinical features (11). However, to our knowledge, the influence of various Iranian ethnicities on clinical patterns has not been studied. The aim of this study was to assess whether clinical measures in AS disease are influenced by various Iranian ethnicities including Fars, Turk, Kord, Lor, etc.

## Methods

From May 2010 to March 2011, a total of 163 Iranian cases with AS aged 16 and older were consecutively enrolled in this study from three sources: 1) Iran AS Association (an association in which the members are AS patients with heterogeneous Iranian ethnic origins), 2) Iran Rheumatology Center and 3) Rheumatology Clinic in Shariati Hospital. Modified New-York criteria 1984 was used for diagnosis (12). The study protocol was compatible with Helsinki declaration (revised 2008) and approved by the Research Ethics Committee of Tehran University of Medical Sciences. A structured questionnaire was applied for gathering information. For providing uniformity, all metrology indices were measured by the same rheumatologist. The metrology indices included: BASMI score (from 0 -the best mobility to 10 -the worst mobility), finger to floor distance (cm), chest expansion (cm), modified Schober (cm), cervical rotation (degree), tragus to wall distance (cm), decrease in cervical slope, increase in thoracic kyphosis, decrease in lumbar lordosis. The Persian version of Bath AS disease activity index (BASDAI), Bath AS functional index (BASFI) and AS quality of life (ASQoL) questionnaires were used for assessing disease activity, functional status and quality of life, respectively (13-17). HLA-B*27 was evaluated by polymerase chain reaction with sequence specific primer (PCR-SSP).

For presenting continuous and categorical variables, mean ± standard deviation (SD) and frequency (%) were used, respectively. For comparing continuous variables between ethnic origins, Kruskal-Wallis test was used. Chi-square where appropriate and Fisher’s exact tests were used for comparing categorical variables. Simple linear regressions models were used for evaluating the relationship between Iranian ethnic origins (as independent variable) and each of the clinical measures (as dependent variable). Fars ethnic origin was considered as reference point. Dummy variable was constructed for each of the Turk, Kord, Lor and other ethnic origins and all regressions were estimated regarding Fars ethnicity as reference point. P-value < 0.05 was considered significant.

## Results

The ethnic origins of the 163 studied patients included: Fars 80 (49.1%), Turk 49 (30.1%), Kord 12 (7.4%), Lor 12 (7.4%) and other ethnicities, 10 (6.1%) individuals. Male to female ratio was 3.79. The age of patients was 37.74±9.88 (range: 18-65) years and disease duration was 14.49±8.47 (range: 1-44) years. The details of demographic and clinical features in all ethnic origins were shown and compared in tables 1 and 2. Finger to floor distance was significantly different between various ethnicities (P=0.043). The highest and the lowest finger to floor distance was observed for Fars ethnicity (20.438±14.784) and other ethnicities (5.90±8.0478), respectively (table 1). 

A significant relationship was not seen between the ethnicities and tragus to wall distance or cervical rotation (P>0.05). However, the frequency of severe decrease in cervical slope was significantly different between various ethnicities (P=0.025). The most and the least frequency of severe decrease in cervical slope was observed in Fars patients (61.3%) and other ethnicities (20%), respectively (table 2). The frequency of total increase in thoracic kyphosis was not significantly different between the ethnicities (P>0.05). However, the frequency of severe thoracic kyphosis was significantly dissimilar between various ethnicities (P=0.006). 

The most and the least frequency of severe increase in thoracic kyphosis was observed in Fars (68.8%) and Lor patients (25%), respectively. After Lor ethnicity, the other ethnicities had the lowest frequency for severe thoracic kyphosis (30%) (table 2). The results of relationship between various Iranian ethnic origins and some clinical measures were summarized in tables 3 and 4. A significant relationship was observed only between other ethnicities and finger to floor distance (P=0.003), lateral lumbar flexion (P=0.03), chest expansion (P=0.03) and BASDAI (P=0.03). No significant correlation was seen between Iranian ethnic origins and other clinical measures including intermalleolar distance, cervical rotation, tragus to wall distance, nocturnal back-pain, total back-pain, modified Schober and ASQoL (P>0.05).

**Table 1:** Comparing continuous clinical and demographic parameters between various Iranian ethnic origins

	**Fars**	**Turk**	**Kord**	**Lor**	**Other ethnicities**	**P-value** *
Age (year)	39.14±9.68	39.69±10.88	34.58±6.04	38.50±9.60	34.60±9.82	0.42
Age at symptom onset (year)	24.43±6.64	21.90±7.64	23.17±5.95	24.67±9.23	21.20±5.29	0.28
Age at diagnosis (year)	33.08±9.45	29.02±10.42	29.33±6.76	33.08±9.90	28.10±8.20	0.13
Disease duration (year)	14.83±8.66	14.82±9.18	12.50±6.86	13.83±6.03	13.40±8.53	0.98
Chest expansion (cm)	4.08±2.02	4.27±1.83	4.33±1.87	4.04±1.54	5.50±2.07	0.22
Finger to floor distance (cm)	20.44±14.78	17.88±13.59	15.17±17.11	16.67±13.93	5.90±8.05	0.04
Modified Schober (cm)	3.21±2.07	3.59±2.00	4.00±2.22	3.42±1.95	4.15±1.47	0.36
Tragus to wall (cm)	18.37±6.78	17.18±5.42	18.50±6.97	17.98±7.65	17.05±6.31	0.74
Cervical rotation (degree)	59.63±23.31	63.08±19.58	68.33±16.83	61.25±20.41	71.25±10.69	0.43
Intermalleolar distance (cm)	95.63±27.14	92.08±23.86	96.92±16.30	102.17±9.03	100.3±20.49	0.62
ASQoL (0-18)	7.95±5.27	8.14±5.33	8.50±5.73	8.75±4.58	6.60±6.02	0.87
BASMI (0-10)	4.22±1.89	3.85±1.87	3.43±2.10	3.78±1.78	3.08±1.63	0.18
BASDAI (0-10)	4.58±2.24	4.76±2.30	4.71±2.69	4.45±2.37	3.12±2.13	0.34
BASFI (0-10)	4.08±2.73	4.28±2.75	4.93±3.18	3.91±2.18	2.10±2.24	0.15
Pack-years of smoking	2.83±6.37	1.15±2.76	2.36±5.83	7.75±18.46	3.00±9.49	0.80
Lateral lumbar flexion (cm)	10.18±6.24	11.20±6.37	13.35±5.73	9.88±5.60	14.65±4.64	0.07

* All p-values were calculated by Kruskal-Wallis test.

**Table 2:** Frequency distribution of clinical and demographic parameters in various Iranian ethnic origins

			**Fars** **N (%)**	**Turk** **N (%)**	**Kord** **N (%)**	**Lor** **N (%)**	**Others** **N (%) **	**P-value** *
Female/male			15/65 (23)	12/37 (32)	4/8 (50)	2/10 (20)	1/9 (11)	> 0.05
IBD**			6 (7.5)	4 (8.2)	1 (8.3)	0	0	> 0.05
Uveitis			13 (16.3)	6 (12.2)	1 (8.3)	1 (8.3)	2 (20)	> 0.05
Arthritis			43 (53.8)	29 (59.2)	5 (41.7)	3 (25)	3 (30)	> 0.05
Family history of AS			21 (26.3)	20 (40.8)	2 (16.7)	4 (33.3)	3 (30)	> 0.05
Associated autoimmune disease			10 (12.5)	4 (8.2)	1 (8.3)	0	1 (10)	> 0.05
Enthesitis			53 (66.3)	31 (63.3)	10 (83.3)	10 (83.3)	5 (50)	> 0.05
Psoriasis			5 (6.3)	1 (2)	0	1 (8.3)	0	> 0.05
Non-steroidal Anti-inflammatory drugs			76 (95)	47 (95.9)	11 (91.7)	11 (91.7)	9 (90)	> 0.05
Number of DMARDs***		0 1 2	19 (28.3)	10 (20.4)	1 (8.3)	3 (25)	3 (30)	> 0.05
			42 (52.5)	20 (40.8)	5 (41.7)	5 (41.7)	4 (40)	
			19 (23.8)	19 (38.8)	6 (50)	4 (33.3)	3 (30)	
Infliximab, Ethanercept or both			12 (15)	4 (8.2)	2 (16.7)	1 (8.3)	0	> 0.05
Sacroiliitis grading	2 3 4		24 (30)	15 (30.6)	4 (33.3)	5 (41.7)	5 (50)	> 0.05
			35 (43.8)	23 (46.9)	8 (66.7)	6 (50)	4 (40)	
			21 (26.2)	11 (22.4)	0	1 (8.3)	1 (10)	
Loss of lumbar lordosis	Severe		48 (60)	30 (61.2)	8 (66.7)	9 (75)	4 (40)	> 0.05
	Non-severe		32 (40)	19 (38.8)	4 (33.3)	3 (25)	6 (60)	
Thoracic Kyphosis	Severe		55 (68.8)	23 (46.9)	6 (50)	3 (25)	3 (30)	0.006
	Non- severe		25 (31.2)	26 (53.1)	6 (50)	9 (75)	7 (70)	
Decrease cervical slope	Severe		49 (61.3)	22 (44.9)	6 (50)	3 (25)	2 (20)	0.025
	Non- severe		31 (38.8)	27 (55.1)	6 (50)	9 (75)	8 (80)	
Educational level	Illiterate		0	1(2)	0	0	0	> 0.05
	Primary school		14 (17.5)	13 (26.5)	2 (16.7)	2 (16.7)	2 (20)	
	Secondary school		1 (1.3)	0	0	1 (8.3)	0	
	High school		29 (36.3)	21 (42.9)	5 (41.7)	4 (33.3)	5 (50)	
	University		36 (45)	14 (28.6)	5 (41.7)	5 (41.7)	3 (30)	
HLA-B27 positive			60 (75)	38 (77.6)	10 (83.3)	7 (58.3)	7 (70)	> 0.05

* All p-values were calculated by chi-square and needed Fisher’s exact.

** Inflammatory bowel disease

*** Disease modifying anti-rheumatic drugs

**Table 3.** Simple linear regressions with various Iranian ethnic origins as independent variable and BASFI, BASDAI, BASMI as dependent variables

	**BASFI**			**BASDAI**			**BASMI**		
	**B**	**SE**	**P-value**	**B**	**SE**	**P-value**	**B**	**SE**	**P-value**
Turk	0.18	0.42	0.66	0.21	0.49	0.68	-0.38	0.34	0.27
Kord	0.13	0.71	0.85	0.86	0.84	0.31	-0.79	0.58	0.18
Lor	-0.13	0.71	0.86	-0.17	0.84	0.84	-0.44	0.58	0.45
Others	-1.46	0.77	0.06	-1.98	0.91	0.03	-1.14	0.63	0.07

Fars ethnic origin was considered as reference point. Dummy variable was constructed for each of the Turk, Kord, Lor and other ethnic origins. All regression coefficients were estimated regarding Fars ethnicity as the reference point.

BASFI: Bath AS functional index, BASDAI: Bath AS disease activity index, BASMI: Bath AS metrology index, B: regression coefficient, SE: standard error

**Table 4:** Simple linear regressions with various Iranian ethnic origins as independent variable and chest expansion, finger to floor distance, lateral lumbar flexion as dependent variables

	**Chest expansion**			**Finger to floor distance**			**Lateral lumbar flexion**		
	**B**	**SE**	**P-value**	**B**	**SE**	**P-value**	**B**	**SE**	**P-value**
Turk	0.18	0.35	0.60	-2.55	2.59	0.33	1.02	1.11	0.36
Kord	0.25	0.60	0.67	-5.27	4.41	0.23	3.17	1.90	0.10
Lor	-0.04	0.60	0.95	-3.77	4.41	0.39	-0.31	1.90	0.87
Others	1.42	0.65	0.03	-14.54	4.78	0.003	4.47	2.05	0.03

Fars ethnic origin was considered as the reference point. Dummy variable was constructed for each of the Turk, Kord, Lor and other ethnic origins. All regression coefficients were estimated regarding Fars ethnicity as the reference point. B: regression coefficient, SE: standard error

## Discussion

The results show that severe increase in thoracic kyphosis and severe decrease in cervical slope are most common in Fars and least common in other ethnic origins. The most and the least important limitations in lumbar flexion as revealed by finger to floor distance were observed in Fars and other ethnic origins, respectively. A significant relationship between other ethnicities and finger to floor distance, lateral lumbar flexion, chest expansion and BASDAI revealed that these measures are also probably influenced by Iranian ethnic origins (tables 3 and 4). 

For those clinical measures in which the significant differences were not observed between various ethnicities, a trend toward more severe disease was seen in Fars patients compared with others. For instance, a trend towards poorer functional status (measured by BASFI) and worse spinal mobility (measured by BASMI) was observed in Fars ethnic origin versus other ethnic origins (table 3). 

Direct comparisons between different ethnic groups in AS patients are not widely available. A nationwide study on Brazilian patients has revealed the association of ethnic background with clinical features of spondyloarthropathies. The African Brazilians had higher decrease in lateral lumbar flexion while the whites had higher occiput to wall distance. Their results showed poorer quality of life and worse disease for the African Brazilian patients compared to the white patients (11). A comparison between the Middle East Arabs (MEA) and South Asians (SA) has been carried out in a small study in Kuwait by Uppal et al. Family history was more common and peripheral arthritis was less common in MEA compared to SA. However, the clinical and functional measures were not reported in their survey (18). As an advantage, the current study is the first study to assess the effect of various Iranian ethnic origins on clinical measures in patients with AS. The small sample size may be the limitation of our study. However, this survey was noted as a pilot study. 

In conclusion, clinical measures might be influenced by various Iranian ethnic origins in AS patients. Therefore, an extended nationwide survey is suggested with inclusion of patients from all Iranian ethnic origins (Fars, Turk, Kurd, Lor, Baluch, Arab, etc) to demonstrate the definite variations in clinical expression for various Iranian ethnic origins. 

## Notes

**Funding**: This study was a part of project, supported and funded by Rheumatology Research Center, Tehran University of Medical Sciences (grant number: 89-03-41-11076).
